# Aspirination of α-Aminoalcohol (Sarpogrelate M1)

**DOI:** 10.3390/molecules21091126

**Published:** 2016-08-25

**Authors:** Sunhwa Park, Jiyun Lee, Kye Jung Shin, Jae Hong Seo

**Affiliations:** Integrated Research Institute of Pharmaceutical Sciences, College of Pharmacy, The Catholic University of Korea, Bucheon-si, Gyeonggi-do 420-743, Korea; sunalovegs@catholic.ac.kr (S.P.); ljiy727@catholic.ac.kr (J.L.); kyejung@catholic.ac.kr (K.J.S.)

**Keywords:** aspirin, sarpogrelate, α-aminoalcohol, esterification

## Abstract

Aspirination of α-aminoalcohol (sarpogrelate M1) has been performed under various general esterification conditions. In most cases, the desired aspirinate ester was obtained at a low yield with unexpected byproducts, the formation of which was mostly derived from the chemical properties of the tertiary α-amino group. After systematic analysis of those methods, the aspirinated sarpogrelate M1 was prepared using a two-step approach combining salicylate ester formation and acetylation.

## 1. Introduction

**Figure 1:**
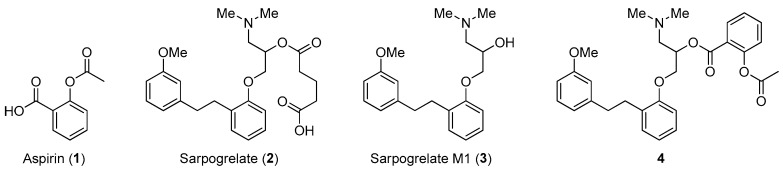
Structure of antiplatelet drugs and metabolites.

Aspirin (**1**) is a representative non-steroidal anti-inflammatory drug (NSAID) used as an analgesic, antipyretic, anti-inflammatory, and antiplatelet drug. Aspirin has also been chemically conjugated with other drugs or biologically active compounds to increase its anti-inflammatory or antiplatelet activity [1,2,3,4,5,6], for other uses such as treating cancer or bacterial infection, or to create biomarkers [7,8,9,10,11]. During our ongoing efforts to identify a novel antiplatelet drug by chemical conjugation of two drugs, we designed an aspirin-hybrid sarpogrelate metabolite 1 (M1) (**4**). Sarpogrelate M1 (**3**) is an active metabolite of sarpogrelate (**2**) [12], which has been clinically used with aspirin in dual or triple antiplatelet therapy to prevent cardiovascular events (Figure 1) [13,14]. We anticipated that simple esterification of aspirin and sarpogrelate M1 would provide the desired aspirinate ester compound, since numerous synthetic methods for aspirinate ester formation from various alcohols have been reported. For example, aspirinyl chloride is considered a useful coupling partner in ester formation [4,7,11]. In addition, other coupling conditions, such as dicyclohexylcarbodiimide (DCC) coupling [5,6,8], carbonyldiimidazole (CDI) activation [3], and Mitsunobu conditions [15,16] have been utilized for the esterification of aspirin and alcohols. However, aspirinate ester formation of α-aminoalcohol is rarely reported. *N*-Boc protected α-aminoalcohol could be aspirinated by treatment with DCC or polymer-supported CDI with aspirin [17]. To the best of our knowledge, there is only one precedent for the aspirination of an alcohol compound having a tertiary amino group at the α-position, in which aspirinic anhydride was used as a coupling reagent to yield the corresponding ester compound at a low yield (19%) [18]. Here, we discuss unexpected byproduct formation during aspirinate ester formation of alcohol compounds with a tertiary α-amino group—represented by sarpogrelate M1 (**3**)—under general esterification conditions, and our efforts to identify an effective synthetic method for the transformation.

## 2. Results and Discussion

To verify the possibility of synthesizing the desired ester **4** under previously reported conditions, we selected four esterification conditions that were previously used for aspirination of other alcohols: DCC coupling [9], CDI activation [3], Mitsunobu [15], and aspirinyl chloride coupling [4]. All results from the reaction of sarpogrelate M1 (**3**) under these conditions are shown in Table 1. To explore the difference between α-aminoalcohol and normal alcohol under each aspirination condition, the same reaction conditions were applied to benzyl alcohol (Table 2).

When sarpogrelate M1 (**3**) was exposed to DCC coupling conditions with aspirin, the desired ester **4** was generated at low yield (13% yield, Table 1, entry 1). Interestingly, the reaction afforded acetate ester **5** as a major product, with 87% yield. Although Fang and coworkers recently reported the formation of acetylated by-products in DCC coupling of aspirin and a quercetin derivative with two phenolic hydroxyl groups, they did not comment on possible reason for these results [19]. Interestingly, comparative experiments with benzyl alcohol yielded aspirinated ester **9** as a major product in 70% yield, as well as a small amount of benzyl acetate (**10**) (17% yield, Table 2, entry 1), which suggests that the tertiary α-amino group of **3** contributes to the acetylation. Increasing the amount of 4-dimethylaminopyridine (DMAP) (0.5 eq vs. 0.1 eq) slightly enhanced production of the acetate ester **5** (89% vs. 87% yield, Table 1, entry 2). For benzyl alcohol, the addition of 0.5 eq of DMAP significantly increased the formation of acetate ester **10** (40% vs. 17% yield) along with the desired aspirinate ester **9** and salicylate ester **11** in 41% and 8% yield, respectively (Table 2, entry 2). These results suggest that DMAP is a major contributor to the formation of acetate esters in the DCC coupling reaction of aspirin, and the tertiary α-amino group of **3** would play a role similar to DMAP in the reaction. This inference is further strengthened by the results of the DCC coupling reaction without DMAP. The absence of DMAP resulted in incomplete reactions for both substrates to recover unreacted **3** (39% yield) and **8** (34% yield), along with coupling products; namely, aspirinate ester **4** (35% yield) and **9** (36% yield) (Table 1 and Table 2, entry 3). Even without DMAP, the reaction of α-aminoalcohol **3** still afforded small amounts of acetate ester **5** (8% yield), which supports the conclusion that the tertiary α-amino group facilitates acetate formation, but not as strongly as DMAP.

Next, CDI activation conditions were applied for the esterification of **3** with aspirin (Table 1, entry 4). To our surprise, the reaction yielded salicylate ester **6** as a sole product at a 75% yield, rather than the expected **4**. Changing the solvent from CH_2_Cl_2_ to CH_3_CN increased the yield of **6** (84% yield) (Table 1, entry 5). The reaction of benzyl alcohol also yielded benzyl salicylate (**11**) as a major product in both solvents (CH_2_Cl_2_: 55% yield, CH_3_CN: 42% yield) along with substantial amounts of the desired aspirinate ester **9** (CH_2_Cl_2_: 15% yield, CH_3_CN: 27% yield) (Table 2, entries 4 and 5). Since small amounts of salicylate esters **6** and **11** were also detected in the DCC coupling reaction (Table 1, entry 3 and Table 2, entry 2), these byproducts may result from the deacetylation of aspirinate esters **4** and **9** by nucleophilic bases; namely, through DMAP or in situ-generated imidazole during CDI activation. To test this hypothesis, aspirinate esters **4** and **9** were exposed to base (1 eq) at room temperature for 12 h (Table 3). For both **4** and **9**, DMAP showed negligible deacetylation (Table 3, entries 1 and 2). In contrast, treatment of **4** with imidazole produced significant amounts of deacetylated compound **6** (Table 3, entries 3 and 4), suggesting that in situ-generated imidazole may cause deacetylation of **4** under CDI activation conditions. In addition, Paradise and coworkers recently reported that imidazole could be used as a selective and mild deacetylating reagent for phenolic acetate [20]. Unlike the case for **4**, the acetyl group of **9** was almost intact when reacted with imidazole (Table 3, entries 5 and 6). Taken together, imidazole does not appear to contribute to deacetylation. In addition, isolation of salicylate dimer **12** indicated that salicylate ester was not produced by simple deacetylation of aspirinate ester, but more likely by direct salicylation. Therefore, during esterification using CDI activation of aspirin, salicylate ester may form as a byproduct, further enhanced by the tertiary α–amino group of the substrate.

**Scheme 1:**
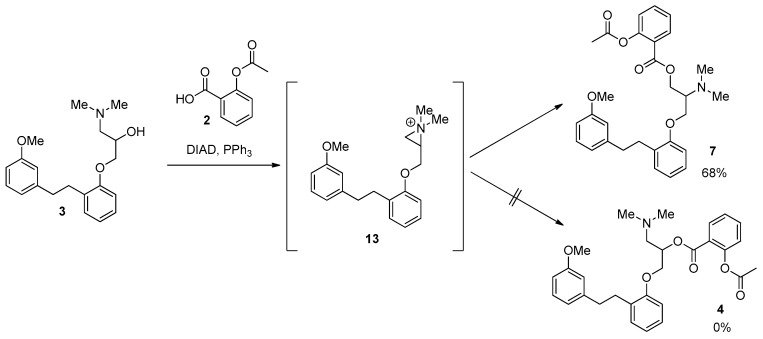
Rearrangement in Mitsunobu reaction of **3**.

Third, Mitsunobu conditions have been used for aspirination of **3** and **8**. Exposure of **8** to Mitsunobu conditions yielded aspirinate ester **9** at a quantitative yield (Table 2, entry 6). However, the reaction with α-aminoalcohol **3** yielded a rearranged product **7** (68% yield), the structure of which was confirmed by 2D-NMR studies including HSQC (heteronuclear single quantum coherence), HMBC (heteronuclear multiple bond correlation), and COSY (correlation spectroscopy) (Table 1, entry 6). This type of rearrangement has been reported in the Mitsunobu reaction of linear and cyclic aminoalcohol substrates [21,22,23]. The rearrangement likely occurs by the generation of an aziridinium intermediate **13**, which is then attacked by the aspirinate anion at the sterically less-hindered carbon to exclusively yield **7** (Scheme 1).

The final aspirination conditions used aspirinyl chloride, which was prepared by treatment with oxalyl chloride and catalytic amounts of DMF. These conditions yielded the desired aspirinate ester **4** in moderate yield (62%, Table 1, entry 7), while benzyl aspirinate **9** was obtained at a relatively low yield (36%) under the same conditions (Table 2, entry 7).

**Scheme 2:**
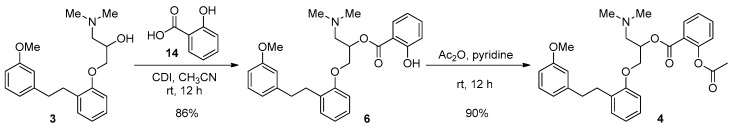
Two-step approach to aspirinate ester **4**.

Despite the somewhat successful results using aspirinyl chloride, we investigated other routes to increase the yield and convenience of the preparation of **4**. We found that the two-step approach was a reliable and efficient synthetic method (Scheme 2). First, instead of aspirin (**2**), salicylic acid (**14**) was used as a coupling partner of **3** under CDI activation conditions to yield salicylate ester **6** (86% yield). The hydroxyl group of **6** was then acetylated by treatment with Ac_2_O and pyridine to yield **4** at a 90% yield. Thus, we could synthesize aspirin-hybrid sarpogrelate M1 **4** through two simple and efficient reactions (salicylation and acetylation) for an overall 77% yield.

## 3. Experimental Section

### 3.1. General Information

All reactions were performed under an argon atmosphere with dry solvents, unless otherwise stated. Dry methylene chloride (CH_2_Cl_2_) tetrahydrofuran (THF) and acetonitrile (CH_3_CN) were obtained from Ultimate Solvent Purification System (JC Meyer Solvent System, Laguna Beach, CA, USA). All commercially available reagents were purchased and used without further purification. Reactions were monitored by thin-layer chromatography (TLC) on silica gel plates (Merck TLC Silica Gel 60 F254, Darmstadt, Germany) using UV light, PMA (an ethanolic solution of phosphomolybdic acid) or ANIS (an ethanolic solution of para-anisaldehyde) as visualizing agent. Purification of products was conducted by column chromatography through silica gel 60 (0.060–0.200 mm). NMR spectra were obtained on Bruker AVANCE III 500 MHz (Bruker Corporation, Billerica, MA, USA) using residual undeuterated solvent or TMS (tetramethylsilane) as an internal reference. High-resolution mass spectra (HR-MS) were recorded on a JEOL JMS-700 (JEOL, Tokyo, Japan) using EI (electron impact).

### 3.2. General Procedures of Esterification and Acetylation of ***6***

#### 3.1.1. DCC Coupling Conditions

To a stirred solution of sarpogrelate M1 **3** or benzyl alcohol (**8**) (0.50 mmol, 1.0 equiv.) in CH_2_Cl_2_ (5 mL) was added aspirin (**1**) (0.55 mmol, 1.1 equiv.), dicyclohexylcarbodiimide (DCC, 0.55 mmol, 1.1 equiv.) and 4-dimethylaminopyridine (equiv. indicated in the text) at 0 °C. Then, the temperature was gradually raised to 25 °C over 30 min. The mixture was stirred at the same temperature for 18 h, and diluted with CH_2_Cl_2_ (50 mL) and sat. aq. NaHCO_3_ (30 mL). The organic layer was separated, dried (Na_2_SO_4_), filtered, and concentrated under reduced pressure. The crude residue was purified by column chromatography (silica gel, hexanes:EtOAc).

#### 3.1.2. CDI Activation Conditions

To a stirred solution of sarpogrelate M1 **3** or benzyl alcohol (**8**) (0.50 mmol, 1.0 equiv.) in CH_2_Cl_2_ (5 mL) or CH_3_CN (5 mL) was added aspirin (**1**) (0.55 mmol, 1.1 equiv.) and 1,1’-carbonyldiimidazole (CDI, 0.60 mmol, 1.2 equiv.) at 25 °C. The mixture was stirred for 12 h, and diluted with CH_2_Cl_2_ (40 mL) and sat. aq. NH_4_Cl (25 mL). The organic layer was separated, dried (Na_2_SO_4_), filtered, and concentrated under reduced pressure. The crude residue was purified by column chromatography (silica gel, hexanes:EtOAc).

#### 3.1.3. Mitsunobu Conditions

To a stirred solution of sarpogrelate M1 **3** or benzyl alcohol (**8**) (0.50 mmol, 1.0 equiv.) in THF (5 mL) was added aspirin (**1**) (0.75 mmol, 1.5 equiv.), triphenylphosphine (0.75 mmol, 1.5 equiv.) and diisopropyl azodicarboxylate (DIAD, 0.75 mmol, 1.5 equiv.) at 0 °C. The mixture was stirred at the same temperature for 1 h, and the solvent was removed under reduced pressure. The residue was diluted with EtOAc (30 mL) and sat. aq. NH_4_Cl (15 mL). The organic layer was separated, dried (Na_2_SO_4_), filtered, and concentrated under reduced pressure. The crude residue was purified by column chromatography (silica gel, hexanes:EtOAc).

#### 3.1.4. Aspirinyl Chloride Coupling Conditions

To a stirred solution of aspirin (**1**) (1.00 mmol, 2.0 equiv.) in CH_2_Cl_2_ (5 mL) was added oxalyl chloride (2 M in CH_2_Cl_2_, 0.60 mL, 1.20 mmol, 2.4 equiv.) and dimethylformamide (DMF, 8.0 μL, 0.10 mmol, 0.2 equiv.) at 0 °C. Then, the temperature was gradually raised to 25 °C. The mixture was stirred at the same temperature for 12 h. Then, to another stirred solution of sarpogrelate M1 **3** or benzyl alcohol (**8**) (0.50 mmol, 1.0 equiv.) in CH_2_Cl_2_ (5 mL) was added pyridine (0.24 mL, 3.0 mmol, 6.0 equiv.) and the previously prepared aspirinyl chloride solution. The mixture was stirred for another 12 h, and diluted with CH_2_Cl_2_ (50 mL) and sat. aq. NaHCO_3_ (30 mL). The organic layer was separated, dried (Na_2_SO_4_), filtered, and concentrated under reduced pressure. The crude residue was purified by column chromatography (silica gel, hexanes:EtOAc).

#### 3.1.5. Acetylation of **6**

To a stirred solution of salicylate ester **6** (241 mg, 0.536 mmol, 1.0 equiv.) in pyridine (2 mL) was added Ac_2_O (76 μL, 0.81 mmol, 1.5 equiv.) at 0 °C. The temperature was raised to 25 °C. The mixture was stirred at the same temperature for 12 h. Then, the mixture was concentrated under reduced pressure and diluted with ethyl acetate (30 mL) and washed with H_2_O (10 mL). The organic layer was separated, dried (Na_2_SO_4_), filtered, and concentrated under reduced pressure. The crude residue was purified by column chromatography (silica gel, hexanes:EtOAc = 1:2) to afford aspirinate ester **4** (239 mg, 90% yield).

*1-(Dimethylamino)-3-(2-(3-methoxyphenethyl)phenoxy)propan-2-yl 2-acetoxybenzoate* (**4**): colorless oil; *R*_f_ = 0.25 (silica gel, hexanes:EtOAc 1:1); ^1^H-NMR (500 MHz, CDCl_3_): δ = 7.99 (dd, *J*_1_ = 1.6 Hz, *J*_2_ = 7.9 Hz, 1H), 7.53–7.50 (m, 1H), 7.18 (ddd, *J*_1_ = 1.1 Hz, *J*_2_ = 7.9 Hz, *J*_3_ = 7.9 Hz, 1H), 7.18–7.14 (m, 2H), 7.10–7.06 (m, 2H), 6.89–6.86 (m, 2H), 6.77 (d, *J* = 7.7 Hz, 1H), 6.72–6.71 (m, 2H), 5.56–5.51 (m, 1H), 4.28–4.22 (m, 2H), 3.75 (s, 3H), 2.92–2.71 (m, 6H), 2.32 (s, 6H), 2.30 (s, 3H) ppm; ^13^C-NMR (125 MHz, CDCl_3_): δ = 169.7, 163.9, 159.7, 156.5, 150.9, 144.1, 134.0, 131.9, 130.5, 130.3, 129.3, 127.4, 126.1, 123.9, 123.4, 121.0, 120.9, 114.2, 111.39, 111.37, 71.2, 67.6, 59.4, 55.2, 46.4, 36.5, 32.8, 21.1 ppm; HRMS (EI): calcd for C_29_H_33_NO_6_ [M^+^]: 491.2308, found 491.2310.

*1-(Dimethylamino)-3-(2-(3-methoxyphenethyl)phenoxy)propan-2-yl acetate* (**5**): colorless oil; *R*_f_ = 0.19 (silica gel, hexanes:EtOAc 1:2); ^1^H-NMR (500 MHz, CDCl_3_): δ = 7.21 (t, *J* = 7.8 Hz, 1H), 7.16 (ddd, *J*_1_ = 1.7 Hz, *J*_2_ = 7.8 Hz, *J*_3_ = 7.8 Hz, 1H), 7.11 (dd, *J*_1_ = 1.7 Hz, *J*_2_ = 7.4 Hz, 1H), 6.87 (ddd, *J*_1_ = 1.0 Hz, *J*_2_ = 7.4 Hz, *J*_3_ = 7.4 Hz, 1H), 6.84 (t, *J* = 8.9 Hz, 2H), 6.78 (t, *J* = 1.9 Hz, 1H), 6.76–6.73 (m, 1H), 5.39–5.34 (m, 1H), 4.19–4.09 (m, 2H), 3.80 (s, 3H), 2.91–2.84 (m, 4H), 2.69–2.61 (m, 2H), 2.30 (s, 6H), 2.05 (s, 3H) ppm; ^13^C-NMR (125 MHz, CDCl_3_): δ = 170.8, 159.7, 156.5, 144.2, 130.5, 130.3, 129.4, 127.4, 121.0, 120.9, 114.3, 111.3, 111.2, 70.4, 67.7, 59.7, 55.3, 46.4, 36.6, 33.2, 21.4 ppm; HRMS (EI): calcd for C_22_H_29_NO_4_ [M^+^]: 371.2097, found 371.2095.

*1-(Dimethylamino)-3-(2-(3-methoxyphenethyl)phenoxy)propan-2-yl 2-hydroxybenzoate* (**6**): colorless oil; *R*_f_ = 0.23 (silica gel, hexanes:EtOAc 2:1); ^1^H-NMR (500 MHz, CDCl_3_): δ = 10.69 (s, 1H), 7.83 (dd, *J*_1_ = 1.7 Hz, *J*_2_ = 8.0 Hz, 1H), 7.44–7.41 (m, 1H), 7.20–7.16 (m, 2H), 7.11 (dd, *J*_1_ = 1.6 Hz, *J*_2_ = 7.4 Hz, 1H), 6.96 (dd, *J*_1_ = 0.9 Hz, *J*_2_ = 8.4 Hz, 1H), 6.91–6.88 (m, 2H), 6.81–6.77 (m, 1H), 6.76–6.72 (m, 3H), 5.70–5.66 (m, 1H), 4.30–4.29 (m, 2H), 3.76 (s, 3H), 2.91–2.82 (m, 6H), 2.40 (s, 6H) ppm; ^13^C-NMR (125 MHz, CDCl_3_): δ = 169.6, 161.8, 159.7, 156.3, 144.0, 136.0, 130.5, 130.4, 130.1, 129.4, 127.4, 121.2, 120.9, 119.4, 117.8, 114.3, 112.5, 111.3, 111.2, 71.2, 67.6, 59.4, 55.2, 46.1, 36.5, 32.8 ppm; HRMS (EI): calcd for C_27_H_31_NO_5_ [M^+^]: 449.2202, found 449.2200.

*2-(Dimethylamino)-3-(2-(3-methoxyphenethyl)phenoxy)propyl 2-acetoxybenzoate* (**7**): colorless oil; *R*_f_ = 0.20 (silica gel, hexanes:EtOAc 1:1); ^1^H-NMR (500 MHz, CDCl_3_): δ = 7.98 (dd, *J*_1_ = 1.6 Hz, *J*_2_ = 7.8 Hz, 1H), 7.55 (ddd, *J*_1_ = 1.7 Hz, *J*_2_ = 7.8 Hz, *J*_3_ = 7.8 Hz, 1H), 7.28 (ddd, *J*_1_ = 1.1 Hz, *J*_2_ = 7.7 Hz, *J*_3_ = 7.7 Hz, 1H), 7.21–7.17 (m, 2H), 7.13 (dd, *J*_1_ = 1.5 Hz, *J*_2_ = 7.4 Hz, 1H), 7.11 (dd, *J*_1_ = 1.0 Hz, *J*_2_ = 8.1 Hz, 1H), 6.92–6.88 (m, 2H), 6.81 (d, *J* = 7.7 Hz, 1H), 6.75–6.73 (m, 2H), 4.62–4.53 (m, 2H), 4.20–4.12 (m, 2H), 3.77 (s, 3H), 3.29–3.24 (m, 1H), 2.95–2.85 (m, 4H), 2.51 (s, 6H), 2.31 (s, 3H) ppm; ^13^C-NMR (125 MHz, CDCl_3_): δ = 169.9, 164.3, 159.7, 156.5, 150.9, 144.0, 134.1, 131.7, 130.4, 130.3, 129.4, 127.4, 126.1, 124.0, 123.2, 121.0, 120.9, 114.2, 111.4, 111.1, 65.7, 62.8, 62.0, 55.2, 42.6, 36.6, 32.6, 21.1 ppm; HRMS (EI): calcd for C_29_H_33_NO_6_ [M^+^]: 491.2308, found 491.2309.

*Benzyl 2-acetoxybenzoate* (**9**) [24]: colorless oil; *R*_f_ = 0.20 (silica gel, hexanes:EtOAc 10:1); 8.08 (dd, *J*_1_ = 1.7 Hz, *J*_2_ = 7.9 Hz, 1H), 7.58–7.55 (m, 1H), 7.45–7.36 (m, 5H), 7.30 (ddd, *J*_1_ = 1.2 Hz, *J*_2_ = 7.7 Hz, *J*_3_ = 7.7 Hz, 1H), 7.10 (dd, *J*_1_ = 1.1 Hz, *J*_2_ = 8.1 Hz, 1H), 5.31 (s, 2H), 2.13 (s, 3H) ppm; ^13^C-NMR (125 MHz, CDCl_3_): δ = 169.8, 164.5, 150.8, 135.6, 134.1, 132.1, 128.8, 128.6, 128.5, 126.2, 124.0, 123.3, 67.2, 20.8 ppm.

*Benzyl acetate* (**10**) [25]: colorless oil; *R*_f_ = 0.33 (silica gel, hexanes:EtOAc 10:1); ^1^H-NMR (500 MHz, CDCl_3_): δ = 7.38–7.32 (m, 5H), 5.11 (s, 2H), 2.11 (s, 3H) ppm; ^13^C-NMR (125 MHz, CDCl_3_): δ = 171.0, 136.1, 128.7, 128.4, 128.3, 66.5, 21.2 ppm.

*Benzyl 2-hydroxybenzoate* (**11**) [26]: colorless oil; *R*_f_ = 0.36 (silica gel, hexanes:EtOAc 20:1); ^1^H-NMR (500 MHz, CDCl_3_): δ = 10.78 (s, 1H), 7.90 (dd, *J*_1_ = 1.7 Hz, *J*_2_ = 8.0 Hz, 1H), 7.48–7.38 (m, 6H), 7.00 (d, *J* = 8.4 Hz, 1H), 6.90–6.87 (m, 1H), 5.40 (s, 2H) ppm; ^13^C-NMR (125 MHz, CDCl_3_): δ = 170.1, 161.9, 135.9, 135.4, 130.1, 128.8, 128.7, 128.4, 119.3, 117.7, 112.5, 67.1 ppm.

*Benzyl 2-((2-hydroxybenzoyl)oxy)benzoate* (**12**): colorless oil; *R*_f_ = 0.26 (silica gel, hexanes:EtOAc 10:1); ^1^H-NMR (500 MHz, CDCl_3_): δ = 10.28 (s, 1H), 8.13 (d, *J* = 7.7 Hz, 1H), 7.99 (d, *J* = 7.9 Hz, 1H), 7.65–7.62 (m, 1H), 7.51 (t, *J* = 7.8 Hz, 1H), 7.40 (t, *J* = 7.7 Hz, 1H), 7.25–7.21 (m, 6H), 7.00 (d, *J* = 8.4 Hz, 1H), 6.91 (t, *J* = 7.6 Hz, 1H), 5.20 (s, 2H) ppm; ^13^C-NMR (125 MHz, CDCl_3_): δ = 169.0, 164.5, 162.1, 149.9, 136.5, 135.2, 134.2, 132.4, 130.7, 128.6, 128.5, 126.7, 124.0, 123.8, 119.6, 117.9, 112.0, 67.5 ppm; HRMS (EI): calcd for C_21_H_16_O_5_ [M^+^]: 348.0998, found 348.0996.

## 4. Conclusions

Aspirinate ester formation of alcohol compound with tertiary α-amino group using general esterification methods was problematic, giving a low yield and byproducts such as acetate ester, salicylate ester, and rearranged products, which seemed to be caused by aspirin itself and the tertiary α-amino group of the substrate. Here, we presented a two-step approach (salicylation and acetylation) as a simple and efficient synthesis method.

## Figures and Tables

**Table 1:**
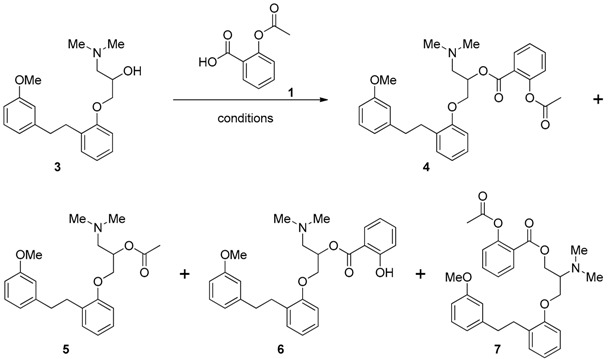
Aspirinate ester formation of sarpogrelate M1 (**3**) under general esterification conditions.

Entry	Conditions	Yield (%) ^1^			
		4	5	6	7
1	**1** (1.1 eq), DCC (1.1 eq), DMAP (0.1 eq), CH_2_Cl_2_, 0 °C, 1 h, then rt, 18 h	13	87	0	0
2	**1** (1.1 eq), DCC (1.1 eq), DMAP (0.5 eq), CH_2_Cl_2_, 0 °C, 1 h, then rt, 18 h	5	89	0	0
3 ^2^	**1** (1.1 eq), DCC (1.1 eq), CH_2_Cl_2_, 0 °C, 1 h, then rt, 18 h	35	8	3	0
4	**1** (1.1 eq), CDI (1.2 eq), CH_2_Cl_2_, rt, 12 h	0	0	75	0
5	**1** (1.1 eq), CDI (1.2 eq), CH_3_CN, rt, 12 h	0	0	84	0
6	**1** (1.5 eq), DIAD (1.5 eq), PPh_3_ (1.5 eq), THF, 0 °C, 1 h	0	0	0	68
7	**1** (2.0 eq), (COCl)_2_ (2.4 eq), DMF (0.2 eq), CH_2_Cl_2_, 0 °C to rt, 12 h; **3**, pyridine (6.0 eq), CH_2_Cl_2_, rt, 12 h	62	0	0	0

^1^ Isolated yield. ^2^ Unreacted **3** (39% yield) has been recovered. CDI: carbonyldiimidazole; DCC: dicyclohexylcarbodiimide; DIAD: diisopropyl azodicarboxylate; DMAP: 4-dimethylaminopyridine; DMF: dimethylformamide; PPh_3_: triphenylphosphine; rt: room temperature.

**Table 2:**
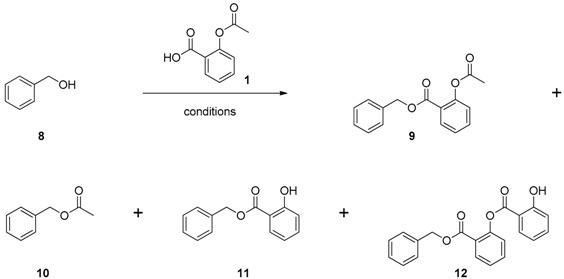
Aspirinate ester formation of benzyl alcohol under general esterification conditions.

Entry	Conditions	Yield (%) ^1^			
		9	10	11	12
1	**1** (1.1 eq), DCC (1.1 eq), DMAP (0.1 eq), CH_2_Cl_2_, 0 °C, 1 h, then rt, 18 h	70	17	0	0
2	**1** (1.1 eq), DCC (1.1 eq), DMAP (0.5 eq), CH_2_Cl_2_, 0 °C, 1 h, then rt, 18 h	41	40	8	0
3 ^2^	**1** (1.1 eq), DCC (1.1 eq), CH_2_Cl_2_, 0 °C, 1 h, then rt, 18 h	36	0	0	0
4	**1** (1.1 eq), CDI (1.2 eq), CH_2_Cl_2_, rt, 12 h	0	15	55	7
5	**1** (1.1 eq), CDI (1.2 eq), CH_3_CN, rt, 12 h	0	27	42	10
6	**1** (1.5 eq), DIAD (1.5 eq), PPh_3_ (1.5 eq), THF, 0 °C, 1 h	100	0	0	0
7	**1** (2.0 eq), (COCl)_2_ (2.4 eq), DMF (0.2 eq), CH_2_Cl_2_, 0 °C to rt, 12 h; **8**, pyridine (6.0 eq), CH_2_Cl_2_, rt, 12 h	36	0	0	0

^1^ Isolated yield. ^2^ Unreacted **8** (34% yield) has been recovered.

**Table 3:**
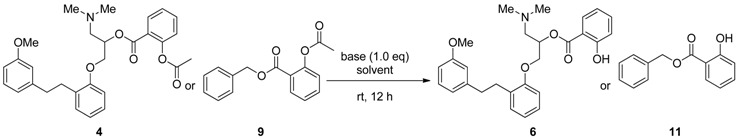
Deacetylation of aspirinate esters **4** and **9** by bases.

Entry	Aspirinate Ester	Base	Solvent	Salicylate Ester	Ratio (4/9:6/11) ^1^
1	**4**	DMAP	CH_2_Cl_2_	**6**	20:1
2	**9**	DMAP	CH_2_Cl_2_	**11**	30:1
3	**4**	imidazole	CH_2_Cl_2_	**6**	3:1
4	**4**	imidazole	CH_3_CN	**6**	2:1
5	**9**	imidazole	CH_2_Cl_2_	**11**	18:1
6	**9**	imidazole	CH_3_CN	**11**	28:1

^1^ Ratio has been determined by ^1^H-NMR of crude mixture.

## Supplementary Materials

The following are available online at: http://www.mdpi.com/1420-3049/21/9/1126/s1, copies of NMR spectra of compounds **4**–**7** and **9**–**12**.
