# Supplementary material for: Aspirination of α-Aminoalcohol (Sarpogrelate M1)
Source: Molecules. 2016 Aug 25;21(9):1126. doi: 10.3390/molecules21091126 (PMC6274198; doi:10.3390/molecules21091126)
Supplement: Supplementary file 1 [file molecules-21-01126-s001.pdf]

Supplementary Materials: Aspirination of α-Aminoalcohol (Sarpogrelate M1)

Sunhwa Park, Jiyun Lee, Kye Jung Shin and Jae Hong Seo

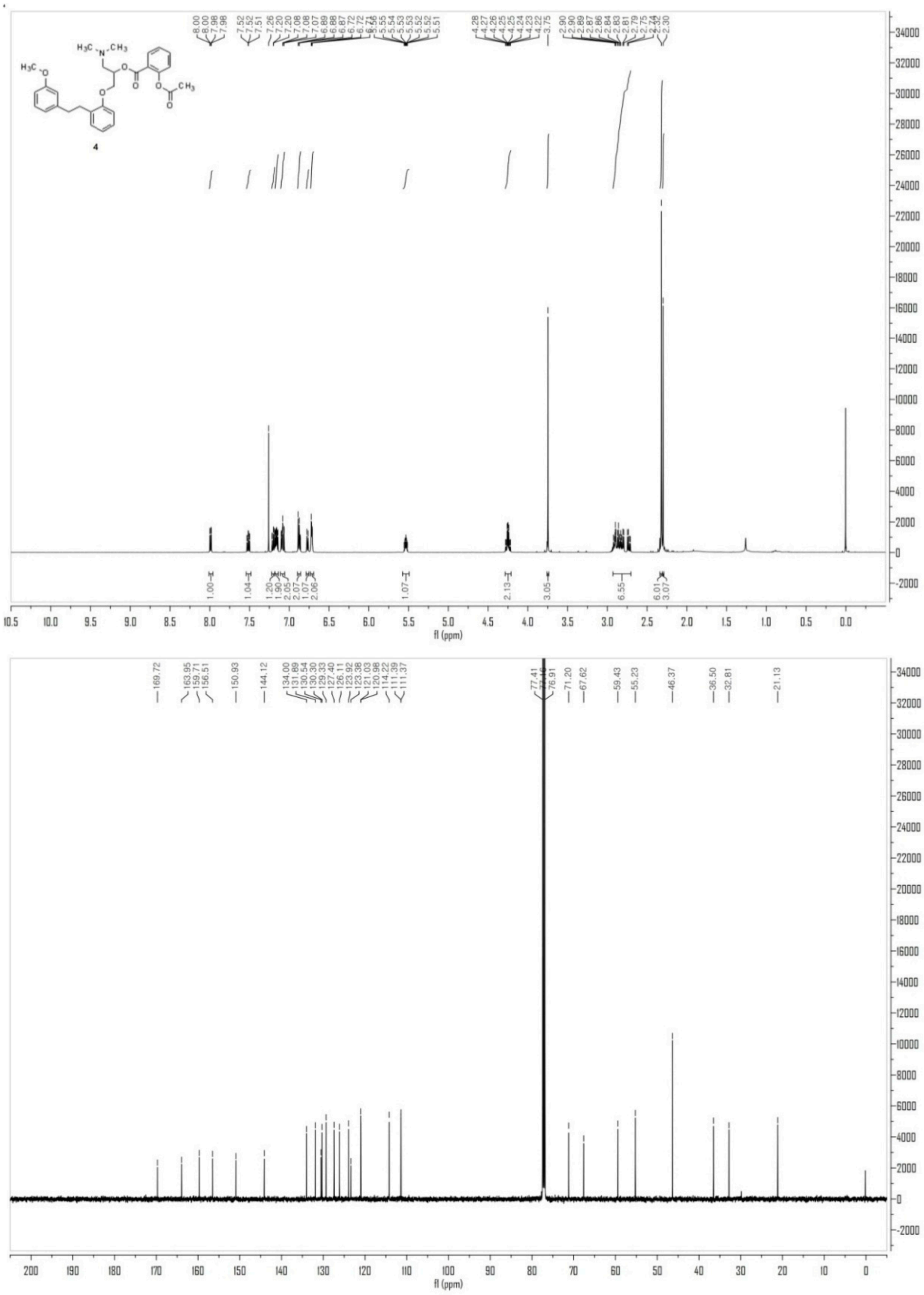

Figure S1. ^{1}H and ^{13}C spectrum of 1-(dimethylamino)-3-(2-(3-methoxyphenethyl)phenoxy) propan-2-yl 2-acetoxybenzoate (**4**).

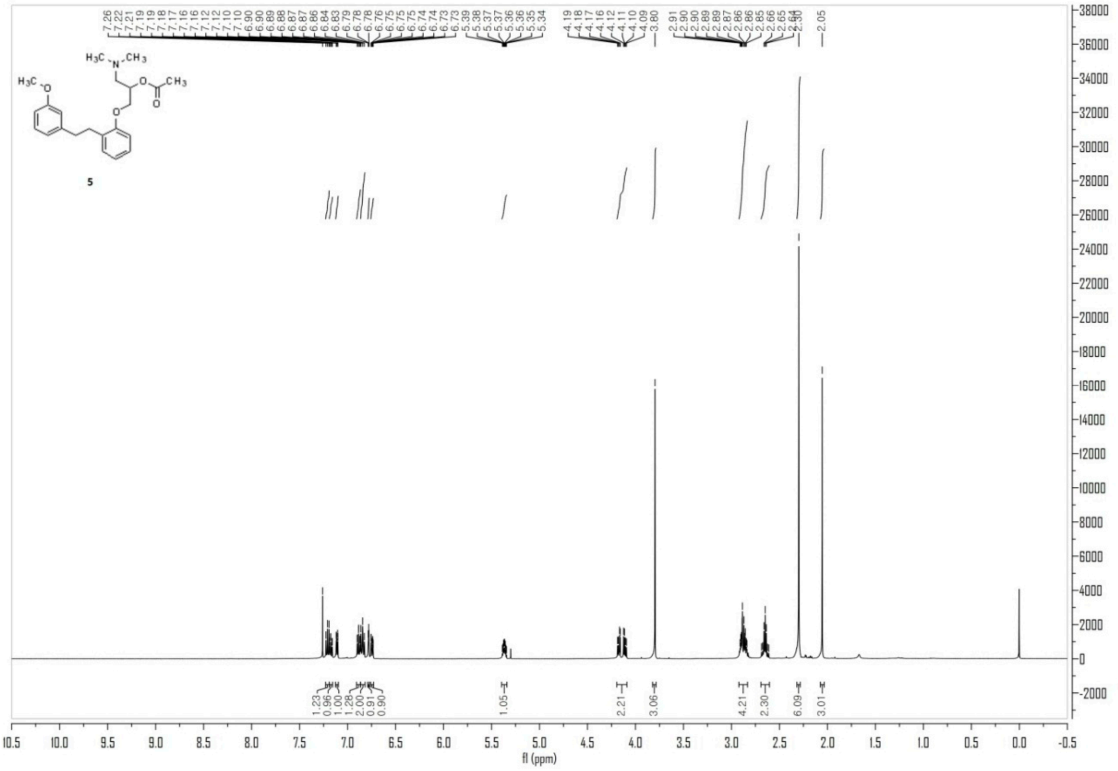

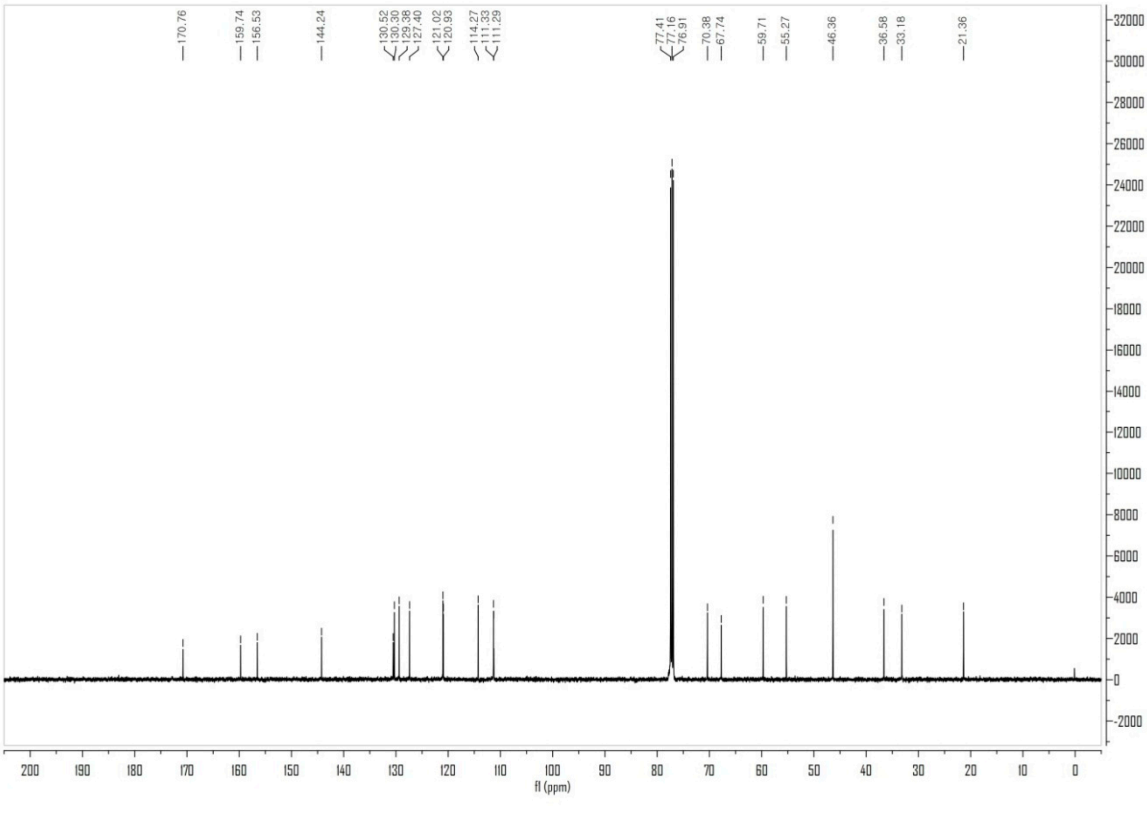

Figure S2. ^{1}H and ^{13}C spectrum of 1-(dimethylamino)-3-(2-(3-methoxyphenethyl)phenoxy) propan-2-yl acetate (**5**).

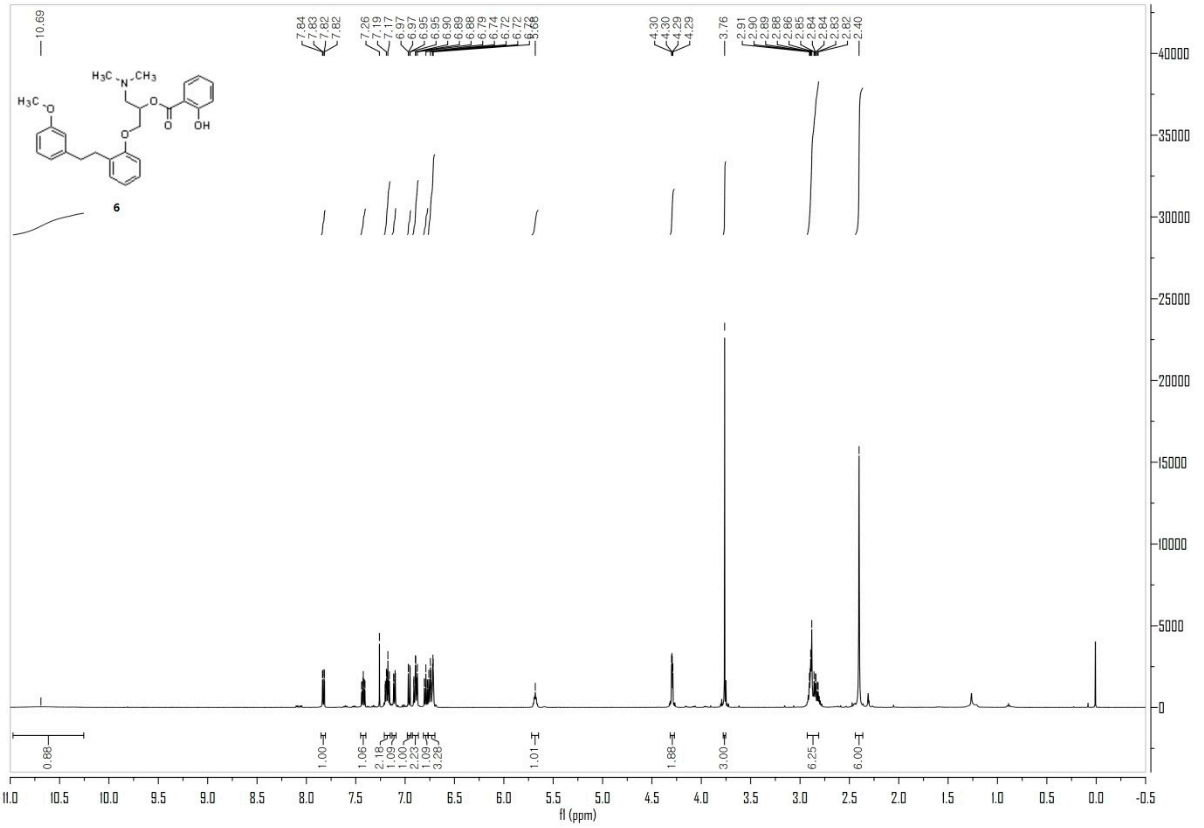

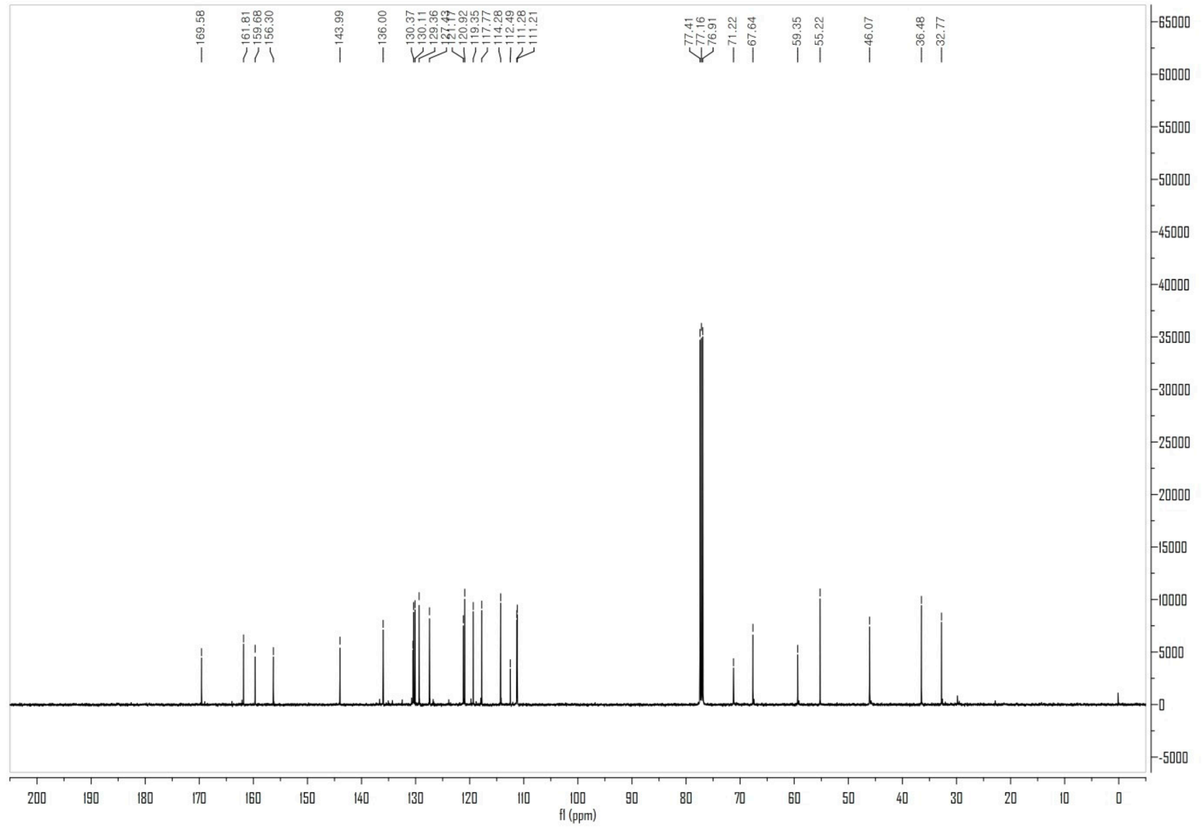

Figure S3. ^{1}H and ^{13}C spectrum of 1-(dimethylamino)-3-(2-(3-methoxyphenethyl)phenoxy) propan-2-yl 2-hydroxybenzoate (**6**).

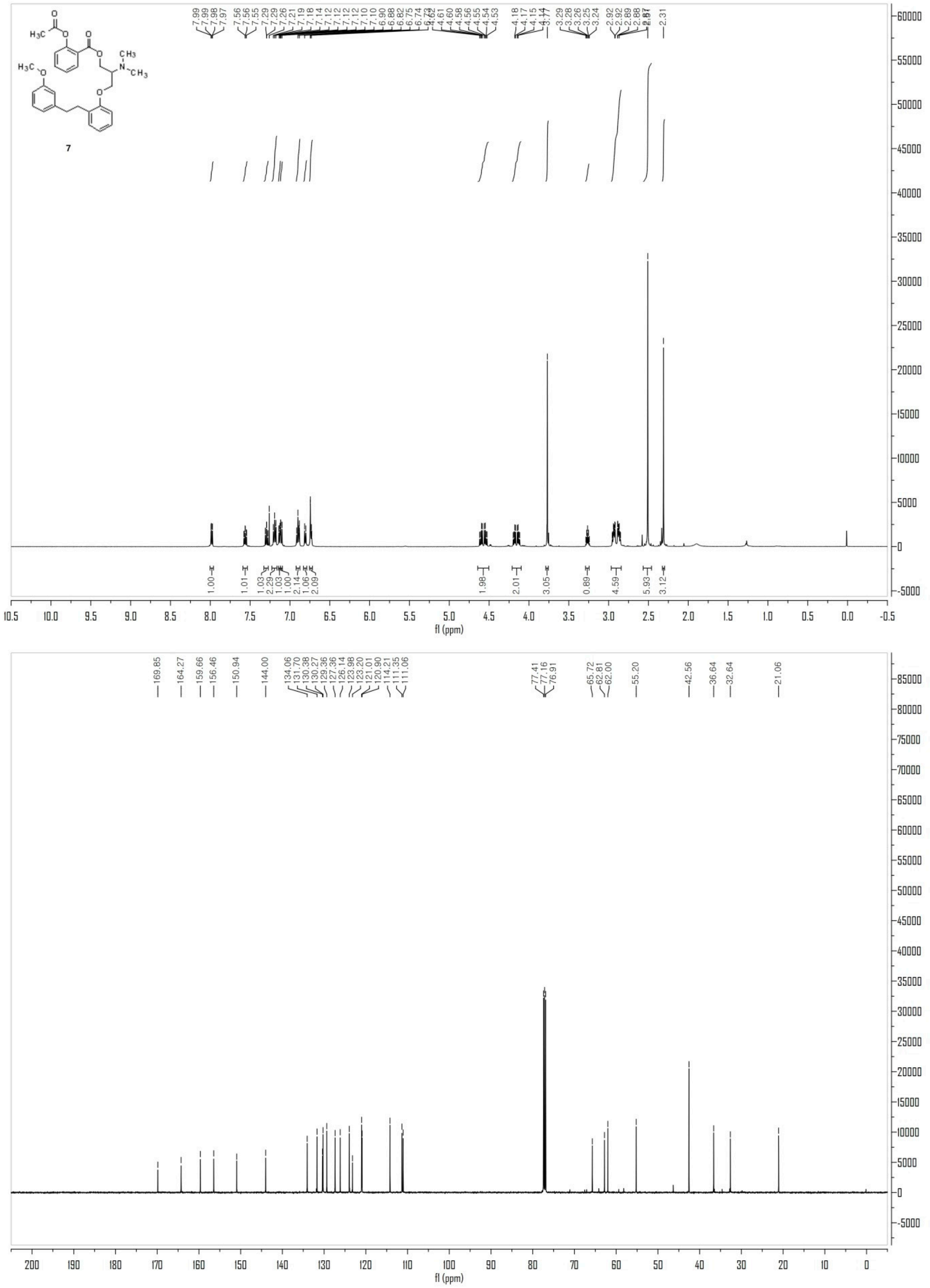

Figure S4. ^{1}H and ^{13}C spectrum of 2-(dimethylamino)-3-(2-(3-methoxyphenethyl)phenoxy) propyl 2-acetoxybenzoate (**7**).

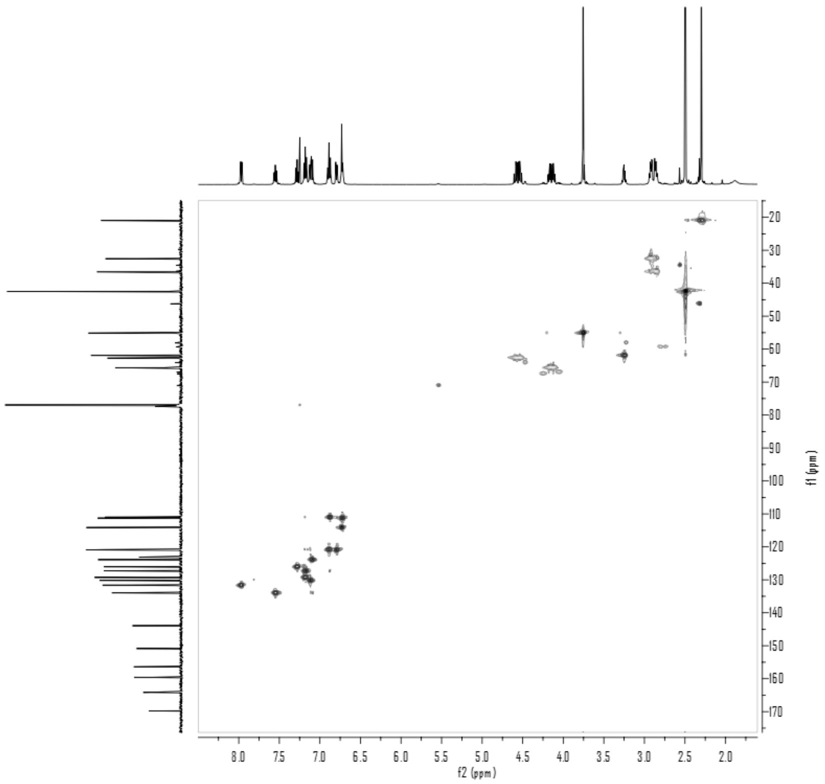

Figure S5. HSQC spectrum of **7**.

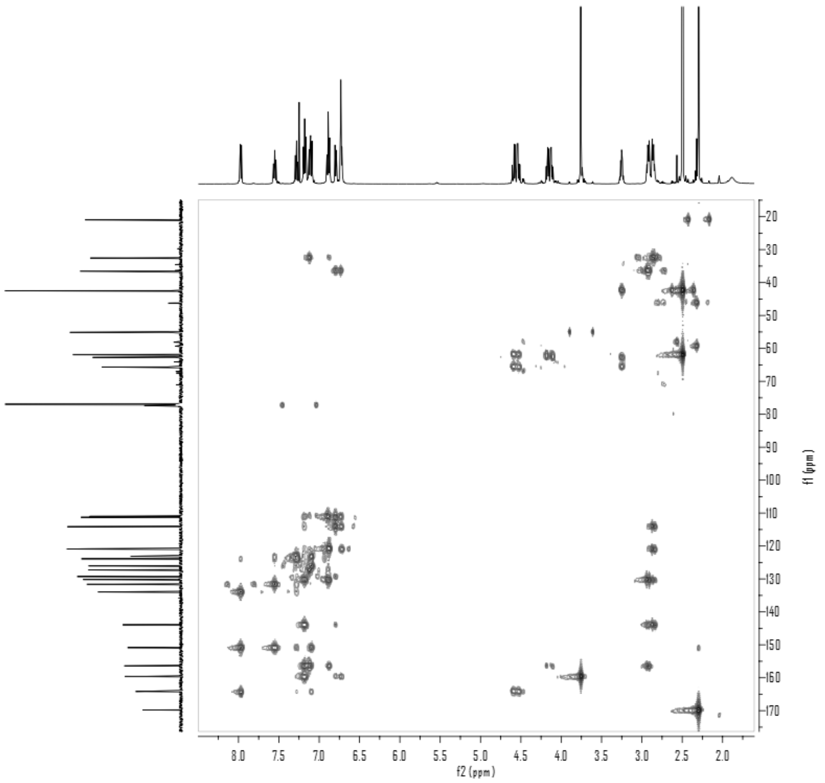

Figure S6. HMBC spectrum of **7**.

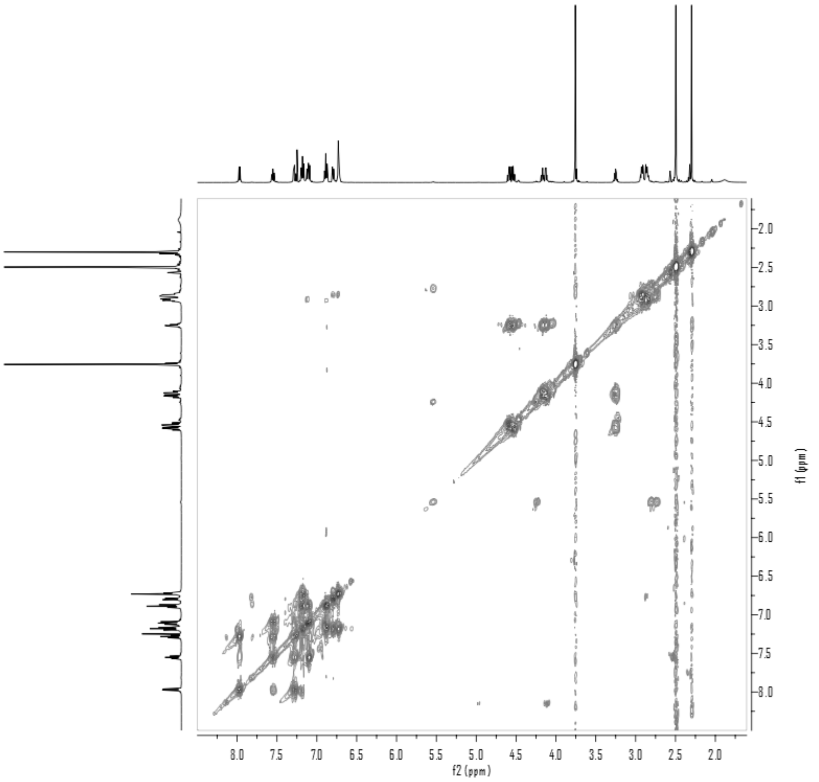

Figure S7. COSY spectrum of **7**.

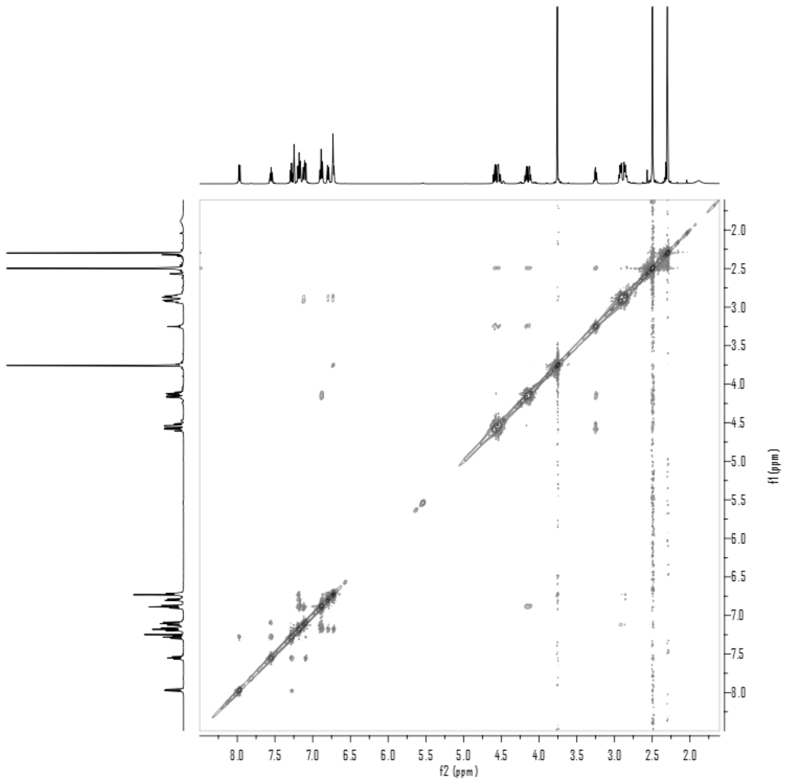

Figure S8. ROESY spectrum of **7**.

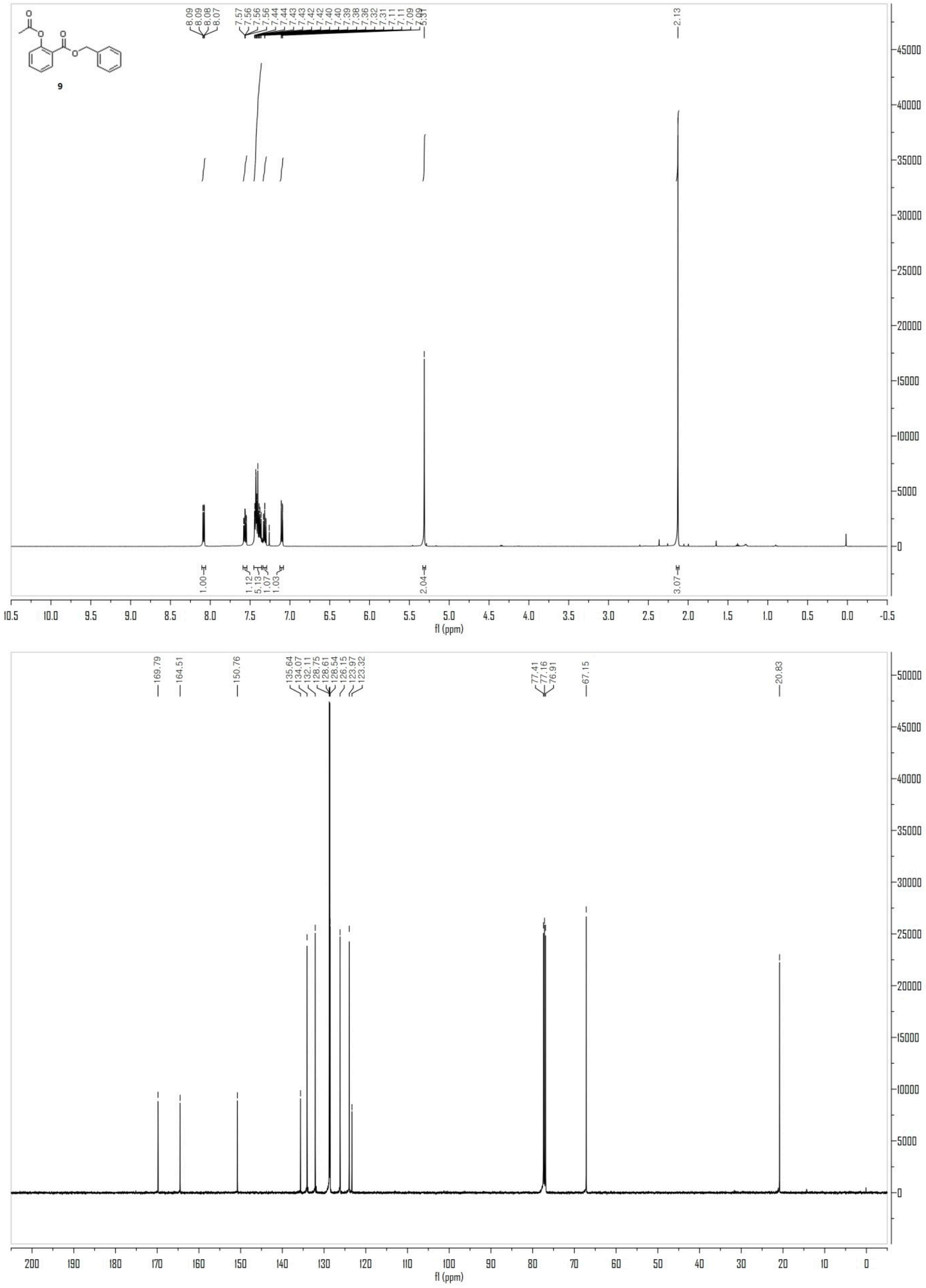

Figure S9. ^{1}H and ^{13}C spectrum of benzyl 2-acetoxybenzoate (**9**).

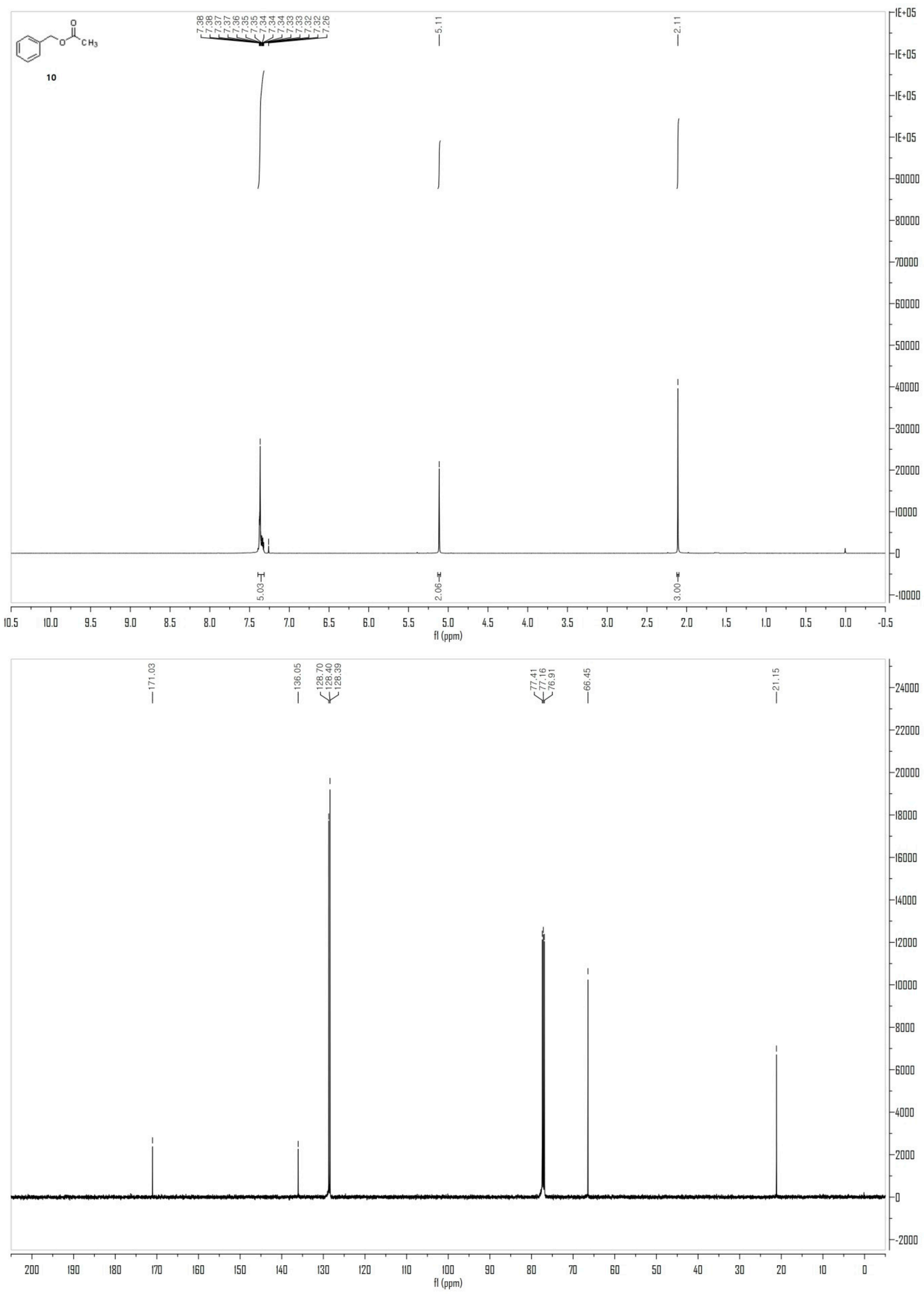

Figure S10. ^{1}H and ^{13}C spectrum of benzyl acetate (**10**).

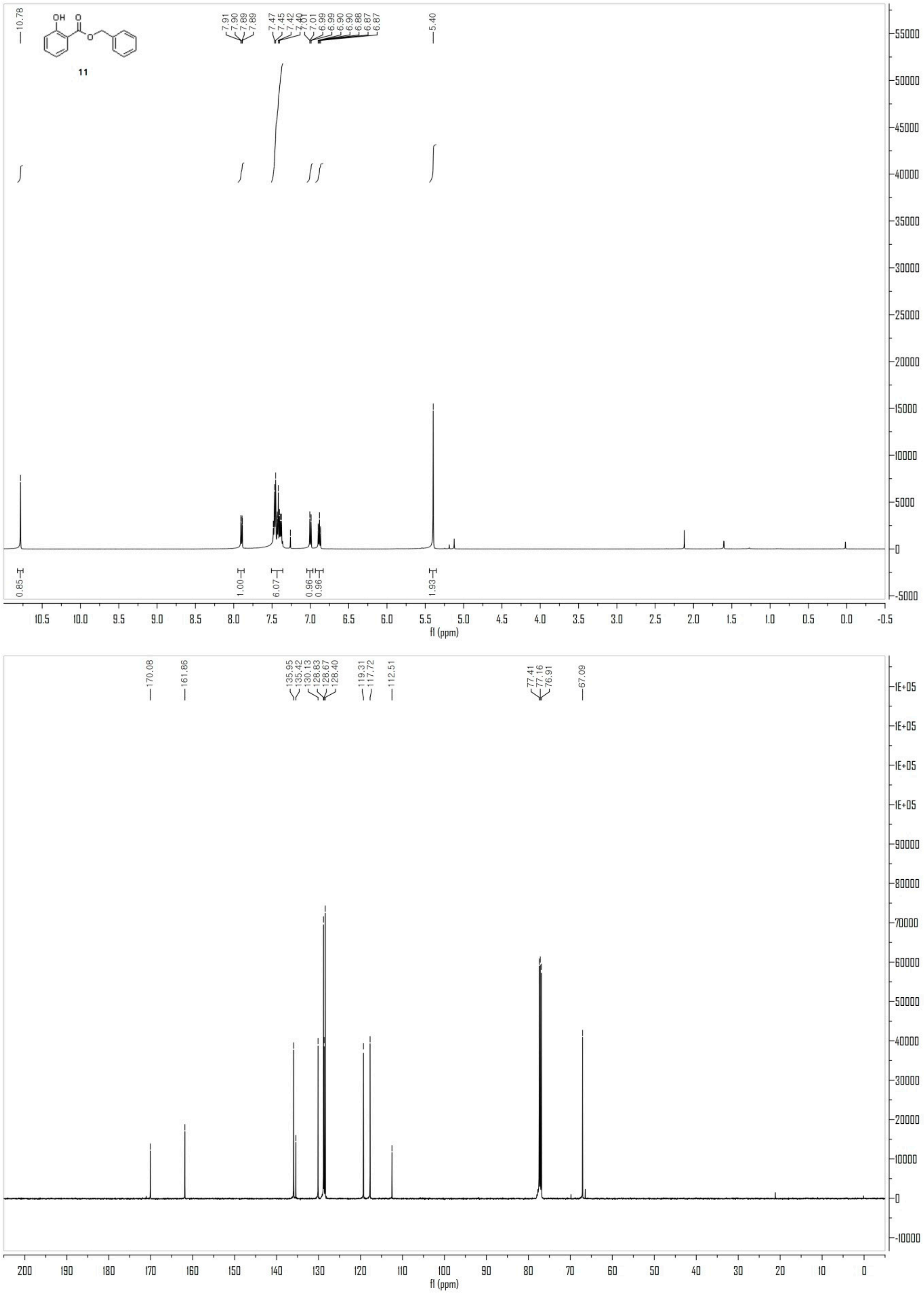

Figure S11. ^{1}H and ^{13}C spectrum of benzyl 2-hydroxybenzoate (**11**).

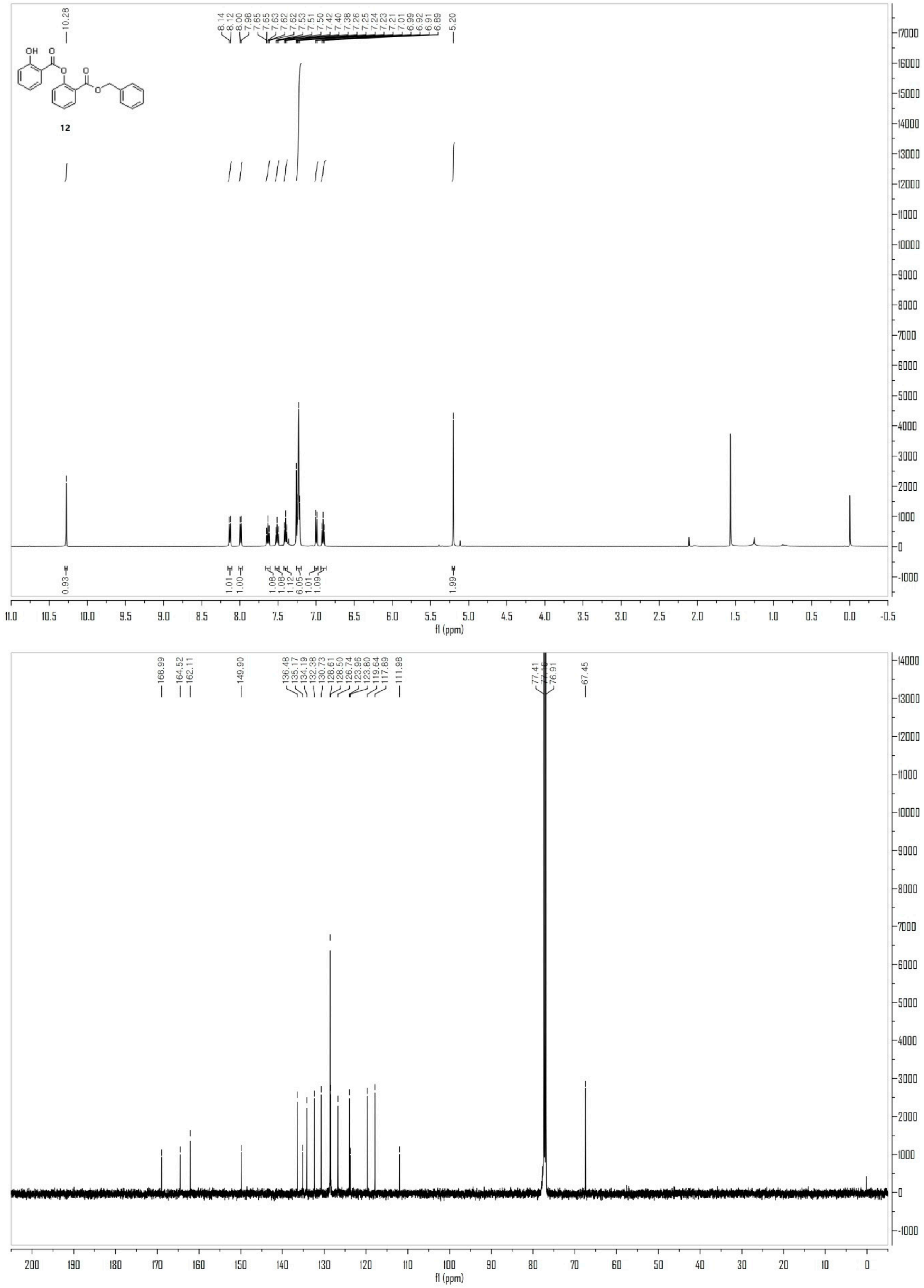

Figure S12. ¹H and ¹³C spectrum of benzyl 2-((2-hydroxybenzoyl)oxy)benzoate (**12**).
